# Supplementary material for: Beyond the revised cardiac risk index: Validation of the hospital frailty risk score in non-cardiac surgery
Source: PLoS One. 2022 Jan 19;17(1):e0262322. doi: 10.1371/journal.pone.0262322 (PMC8769314; doi:10.1371/journal.pone.0262322)
Supplement: S1 Table — (DOCX) [file pone.0262322.s001.docx]

**S1 Table. Procedure codes for non-cardiac surgery.**

| **Surgical Category** | **Surgery Name** | **CCI Code** |
| --- | --- | --- |
| Vascular | Carotid endarterectomy | 1JE57LA |
|  | AAA repair* | 1KA80LA |
|  | Aortofemoral bypass* | 1KA76MZ |
|  | Femoral-popliteal bypass | 1KG76MI |
|  | AV fistula repair | 1KY76LA |
| Abdominal | Gastrectomy | 1NF87DG, 1NF82RJ, 1NF87DH, 1NF87DJ, 1NF87DL, 1NF87DQ, 1NF87LA, 1NF87RG, 1NF87RH, 1NF87RJ, 1NF87RK, 1NF87RP, 1NF87SH |
|  | Total gastrectomy* | 1NF89DZ, 1NF89GW, 1NF89SG, 1NF89TH, 1NF91RG, 1NF91RJ, 1NF91RP, 1NF91SG |
|  | Resection of small intestine* | 1NK87DA, 1NK87DN, 1NK87DP, 1NK87DX, 1NK87DY, 1NK87LA, 1NK87RE, 1NK87RF, 1NK87TF, 1NK87TG |
|  | Partial colectomy* | 1NM87 |
|  | Total colectomy | 1NM89, 1NM91 |
|  | Bowel obstruction* | 1NP72 |
|  | Appendectomy* | 1NV89 |
|  | Splenectomy* | 1OB87, 1OB89 |
|  | Pancreatectomy* | 1OJ87, 1OK89 |
|  | Nephrectomy | 1PC87, 1PC89, 1PC91 |
|  | Cystectomy | 1PM57,1PM80, 1PM84, 1PM87, 1PM89, 1PM90, 1PM91, 1PM92 |
|  | Cholecystectomy | 1OD52, OD57, 1OD80, 1OD89 |
|  | Hysterectomy | 1RM87, 1RM89, 1RM 89 |
|  | Lysis of abdominal adhesions | 1OT72 |
| Thoracic | Lobectomy* | 1GR87, 1GR89, 1GR91, 1GT87 |
|  | Pneumonectomy* | 1GR89, 1GT89, 1GT91 |
| Pelvic | Nephrectomy | 1PM87, 1PM89, 1PM91 |
|  | Prostatectomy | 1QT91 |
|  | Oophorectomy | 1RB57, 1RB87, 1RB89 |
|  | Salpingo-oopherectomy | 1RD89 |
|  | Hysterectomy | 1RM89, 1RM91 |
| Orthopedic | Spinal vertebral repair | 1SC80PF |
|  | Discectomy | 1SE89PF |
|  | Spinal fusion | 1SC75PFNWA |
|  | Below-knee amputation | 1VQ93LA |
|  | Above-knee amputation | 1VC93LA |
|  | Metatarsal amputation | 1WJ93LA |
|  | ORIF femur | 1VC74 |
|  | Knee arthroplasty | 1VG53, 1VG80 |
|  | Hip arthroplasty | 1VA53, 1VA74 |
|  | Ankle ORIF | 1WA74LANW, 1WA73LA, 1WA80 |
|  | Rotator cuff repair | 1TC80, 1TV80 |
|  | Fixation of fracture, radius and ulna | 1TV74 |
|  | Fixation of fracture, tibia and fibular | 1VQ74 |
|  | Cruciate ligament repair | 1VL80 |
| Minor | GI endoscopic | 1NA56BA, 1NE50BA, 1NF13BA, 1NM50BA, 1NM56BA, 1NQ56BA, 2NA70BA, 2NA71BP, 2NA71BR, 2NC70BA, 2NF70BA, 2NF71BA, 2NK70BA, 2NK71BA, 2NK71BR, 2NM70BA, 2NM71BA, 2NM71BR, 2NQ70BA, 2NQ71BA |
|  | Cystoscopy, TURP, TURBT | 1PE56BA, 1PL72BA, 1PM56BA, 1PM87BA, 1PQ50BA, 1PQ56BA, 1QT87BA, 2PG70DA, 2PG71BA, 2PG71DA, 2PG71BR, 2PM70BA, 2PM71BA, 2PM71DA, 2PQ70BA, 2PQ71BA, 2QT70BA, 2QT71BA |
|  | Cataract | 1CL89VRLM |
|  | Mastectomy and superficial procedures | 1YA, 1YB, 1YC, 1YD, 1YE, 1YF, 1YG, 1YH, 1YI, 1YJ, 1JK, 1YL, 1YM, 1YN, 1YO, 1YP, 1YQ, 1YR, 1YS, 1YT, 1YU, 1YV, 1YW, 1YX, 1YY, 1YZ |
|  | Bronchoscopy | 1GJ56BA, 1GM56BA, 1GT56DA, 2GM70BA, 2GM71BA, 2GM71BR, 2GT71BA, 2GT71BP |
|  | Abdominal wall hernia repair | 1SY80 |
