# Supplementary material for: Beyond the revised cardiac risk index: Validation of the hospital frailty risk score in non-cardiac surgery
Source: PLoS One. 2022 Jan 19;17(1):e0262322. doi: 10.1371/journal.pone.0262322 (PMC8769314; doi:10.1371/journal.pone.0262322)
Supplement: S2 Table — (DOCX) [file pone.0262322.s002.docx]

**S2 Table. Hospital frailty risk score weights by ICD code.**

| **ICD Code** | **ICD Description** | **Points awarded** |
| --- | --- | --- |
| **F00** | Dementia in Alzheimer’s disease | 7.1 |
| **G81** | Hemiplegia | 4.4 |
| **G30** | Alzheimer’s disease | 4.0 |
| **I69** | Sequelae of cerebrovascular disease | 3.7 |
| **R29/R29.6** | Other symptoms and signs involving the nervous and musculoskeletal systems | 3.6 |
| **N39** | Other disorders of urinary systems (included urinary tract infection and urinary incontinence) | 3.2 |
| **F05** | Delirium, not induced by alcohol and other psychoactive substances | 3.2 |
| **W19** | Unspecified fall | 3.2 |
| **S00** | Superficial injury of hear | 3.2 |
| **R31** | Unspecified hematuria | 3.0 |
| **B96** | Other bacterial agents as the cause of disease classified to other chapters | 2.9 |
| **R41** | Other symptoms and signs involving cognitive functions and awareness | 2.7 |
| **R26** | Abnormalities of gait and mobility | 2.6 |
| **I67** | Other cerebrovascular diseases | 2.6 |
| **R56** | Convulsions, not elsewhere classified | 2.6 |
| **R40** | Somnolence, stupor and coma | 2.5 |
| **T83** | Complications of genitourinary prosthetic devices, implants and grafts | 2.4 |
| **S06** | Intracranial injury | 2.4 |
| **S42** | Fracture of shoulder and upper arm | 2.3 |
| **E87** | Other disorders of fluid, electrolyte and acid-base balance | 2.3 |
| **M25** | Other joint disorders, not elsewhere classified | 2.3 |
| **E86** | Volume depletion | 2.3 |
| **R54** | Senility | 2.2 |
| **Z50** | Care involving use of rehabilitation procedures | 2.1 |
| **F03** | Unspecified dementia | 2.1 |
| **W18** | Other fall on same level | 2.1 |
| **Z75** | Problems related to medical facilities and other healthcare | 2.0 |
| **F01** | Vascular dementia | 2.0 |
| **S80** | Superficial injury of lower leg | 2.0 |
| **L03** | Cellulitis | 2.0 |
| **H54** | Blindness and low vision | 1.9 |
| **E53** | Deficiency of other B group vitamins | 1.9 |
| **Z60** | Problems related to social environment | 1.8 |
| **G20** | Parkinson’s disease | 1.8 |
| **R55** | Syncope and collapse | 1.8 |
| **S22** | Fracture of rib(s), sternum and thoracic spine | 1.8 |
| **K59** | Other functional intestinal disorders | 1.8 |
| **N17** | Acute renal failure | 1.8 |
| **L89** | Decubitus ulcer | 1.7 |
| **Z22** | Carrier of infectious disease | 1.7 |
| **B95** | Streptococcus and staphylococcus as the cause of diseases classified to other chapters | 1.7 |
| **L97** | Ulcer of lower limb, not elsewhere classified | 1.6 |
| **R44** | Other symptoms and signs involving general sensations and perceptions | 1.6 |
| **K26** | Duodenal ulcer | 1.6 |
| **I95** | Hypotension | 1.6 |
| **N19** | Unspecified renal failure | 1.6 |
| **A41** | Other septicaemia | 1.6 |
| **Z87** | Personal history of other disease and conditions | 1.5 |
| **J96** | Respiratory failure, not elsewhere classified | 1.5 |
| **X59** | Exposure to unspecified factors | 1.5 |
| **M19** | Other arthrosis | 1.5 |
| **G40** | Epilepsy | 1.5 |
| **M81** | Osteoporosis without pathological fracture | 1.4 |
| **S72** | Fracture of femur | 1.4 |
| **S32** | Fracture of lumbar spine and pelvis | 1.4 |
| **E16** | Other disorders of pancreatic internal secretions | 1.4 |
| **R94** | Abnormal results of functions studies | 1.4 |
| **N18** | Chronic renal failure | 1.4 |
| **R33** | Retention of urine | 1.3 |
| **R69** | Unknown and unspecified causes of morbidity | 1.3 |
| **N28** | Other disorders of kidney and ureter, not elsewhere classified | 1.3 |
| **R32** | Unspecified urinary incontinence | 1.2 |
| **G31** | Other degenerative diseases of nervous system, not elsewhere classified | 1.2 |
| **Y95** | Nosomial condition | 1.2 |
| **S09** | Other and unspecified injuries of head | 1.2 |
| **R45** | Symptoms and signs involving emotional state | 1.2 |
| **G45** | Transient cerebral ischaemic attacks and related syndromes | 1.2 |
| **Z74** | Problems related to care provider dependency | 1.1 |
| **M79** | Other soft tissue disorders, not elsewhere classified | 1.1 |
| **W06** | Fall involving bed | 1.1 |
| **S01** | Open wound of head | 1.1 |
| **A04** | Other bacterial intestinal infections | 1.1 |
| **A09** | Diarrhoea and gastroenteritis of presumed infectious origin | 1.1 |
| **J18** | Pneumonia, organism unspecified | 1.1 |
| **J69** | Pneumonitis due to solids and liquids | 1.0 |
| **R47** | Speech disturbances, not elsewhere defined | 1.0 |
| **E55** | Vitamin D deficiency | 1.0 |
| **Z93** | Artificial opening status | 1.0 |
| **R02** | Gangrene, not elsewhere classified | 1.0 |
| **R63** | Symptoms and signs concerning food and fluid intake | 0.9 |
| **H91** | Other hearing loss | 0.9 |
| **W10** | Fall on and from stairs and steps | 0.9 |
| **W01** | Fall on same level from slipping, tripping and stumbling | 0.9 |
| **E05** | Thyrotoxicosis [hyperthyroidism] | 0.9 |
| **M41** | Scoliosis | 0.9 |
| **R13** | Dysphagia | 0.8 |
| **Z99** | Dependence on enabling machines and devices | 0.8 |
| **U80** | Agent resistant to penicillin and related antibiotics | 0.8 |
| **M80** | Osteoporosis with pathological fracture | 0.8 |
| **K92** | Other diseases of digestive system | 0.8 |
| **I63** | Cerebral infarction | 0.8 |
| **N20** | Calculus of kidney and ureter | 0.7 |
| **F10** | Mental and behavioural disorders due to use of alcohol | 0.7 |
| **Y84** | Other medical procedures as the cause of abnormal reaction of the patient | 0.7 |
| **R00** | Abnormalities of heart beat | 0.7 |
| **J22** | Unspecified acute lower respiratory infection | 0.7 |
| **Z73** | Problems related to life-management difficulty | 0.6 |
| **R79** | Other abnormal findings of blood chemistry | 0.6 |
| **Z91** | Personal history of risk-factors, not elsewhere classified | 0.5 |
| **S51** | Open wound of forearm | 0.5 |
| **F32** | Depressive episode | 0.5 |
| **M48** | Spinal stenosis | 0.5 |
| **E83** | Disorders of mineral metabolism | 0.4 |
| **M15** | Polyarthrosis | 0.4 |
| **D64** | Other anaemias | 0.4 |
| **L08** | Other local infections of skin and subcutaneous tissue | 0.4 |
| **R11** | Nausea and vomiting | 0.3 |
| **K52** | Other noninfective gastroenteritis and colitis | 0.3 |
| **R05** | Fever of unknown origin | 0.1 |
