# Supplementary material for: Beyond the revised cardiac risk index: Validation of the hospital frailty risk score in non-cardiac surgery
Source: PLoS One. 2022 Jan 19;17(1):e0262322. doi: 10.1371/journal.pone.0262322 (PMC8769314; doi:10.1371/journal.pone.0262322)
Supplement: S3 Table — (DOCX) [file pone.0262322.s003.docx]

**S3 Table. ELOS by Surgery Type.**

|  | **Surgery Type** | **ELOS (in days)** | **FREQ** |
| --- | --- | --- | --- |
| 1 | Vascular Surgery | 8.1 | 8847 |
| 2 | Abdominal Surgery | 4.8 | 246057 |
| 3 | Thoracic Surgery | 6.3 | 9544 |
| 4 | Pelvic Surgery | 2.4 | 92016 |
| 5 | Orthopedic Surgery | 4.9 | 235554 |
| 6 | Minor Surgery | 8.4 | 348713 |
| 7 | Carotid endarterectomy | 4 | 2883 |
| 8 | AAA repair | 10.7 | 1589 |
| 9 | Aortofemoral bypass | 9.8 | 1080 |
| 10 | Femoral-popliteal bypass | 9.6 | 3014 |
| 11 | AV fistula repair | 13.4 | 399 |
| 12 | Gastrectomy | 14.8 | 1217 |
| 13 | Total gastrectomy | 16.4 | 343 |
| 14 | Resection of small intestine | 15.8 | 11769 |
| 15 | Partial colectomy | 10.8 | 28020 |
| 16 | Total colectomy | 20.7 | 1751 |
| 17 | Bowel obstruction | 13 | 6344 |
| 18 | Appendectomy | 2.8 | 53805 |
| 19 | Splenectomy | 12.9 | 1703 |
| 20 | Pancreatectomy | 13.3 | 1481 |
| 21 | Nephrectomy | 4.9 | 7593 |
| 22 | Cystectomy | 4 | 19145 |
| 23 | Cholecystectomy | 4.2 | 48659 |
| 24 | Hysterectomy | 2.1 | 64902 |
| 25 | Lysis of abdominal adhesions | 6.8 | 24063 |
| 26 | Lobectomy | 6.1 | 9167 |
| 27 | Pneumonectomy | 6.6 | 2186 |
| 28 | Nephrectomy | 3.4 | 14321 |
| 29 | Prostatectomy | 2.4 | 7968 |
| 30 | Oophorectomy | 2.5 | 6946 |
| 31 | Salpingo-oopherectomy | 2.7 | 32881 |
| 32 | Hysterectomy | 2.1 | 57798 |
| 33 | Spinal vertebral repair | 4.9 | 7184 |
| 34 | Discectomy | 3.1 | 840 |
| 35 | Spinal fusion | 6.4 | 4644 |
| 36 | Below-knee amputation | 23.2 | 3548 |
| 37 | Above-knee amputation | 25.9 | 1586 |
| 38 | Metatarsal amputation | 17.5 | 2654 |
| 39 | ORIF femur | 9.8 | 19791 |
| 40 | Knee arthroplasty | 3.1 | 67865 |
| 41 | Hip arthroplasty | 5.6 | 67244 |
| 42 | Ankle ORIF | 3.3 | 25776 |
| 43 | Rotator cuff repair | 1.7 | 8451 |
| 44 | Fixation of FRU | 3.3 | 14894 |
| 45 | Fixation of FTF | 5.6 | 12066 |
| 46 | Cruciate ligament repair | 1.8 | 4326 |
| 47 | GI endoscopic | 10.3 | 120176 |
| 48 | Cystoscopy, TURP, TURBT | 3.8 | 64999 |
| 49 | Cataract | 2.8 | 2225 |
| 50 | Mastectomy and superficial | 9.4 | 107124 |
| 51 | Bronchoscopy | 16.2 | 29042 |
| 52 | Abdominal wall hernia repair | 6.2 | 38876 |
