# Supplementary material for: Beyond the revised cardiac risk index: Validation of the hospital frailty risk score in non-cardiac surgery
Source: PLoS One. 2022 Jan 19;17(1):e0262322. doi: 10.1371/journal.pone.0262322 (PMC8769314; doi:10.1371/journal.pone.0262322)
Supplement: S4 Table — (DOCX) [file pone.0262322.s004.docx]

**S4 Table. Breakdown of HFRS by Surgery.**

|  | **Low risk (<5)** | **Mediate risk (5-15)** | **High risk (>15)** | **Overall** | **p-value** |
| --- | --- | --- | --- | --- | --- |
| Carotid endarterectomy | 2207 (0.4%) | 377 (0.5%) | 44 (0.2%) | 2628 (0.4%) | <.0001 |
| AAA repair* | 1255 (0.2%) | 240 (0.3%) | 12 (0.1%) | 1507 (0.2%) | <.0001 |
| Aortofemoral bypass* | 836 (0.1%) | 156 (0.2%) | 15 (0.1%) | 1007 (0.1%) | <.0001 |
| Femoral-popliteal bypass | 2080 (0.3%) | 372 (0.5%) | 57 (0.3%) | 2509 (0.4%) | <.0001 |
| AV fistula repair | 123 (0.0%) | 145 (0.2%) | 50 (0.2%) | 318 (0.0%) | <.0001 |
| Gastrectomy | 750 (0.1%) | 192 (0.3%) | 25 (0.1%) | 967 (0.1%) | <.0001 |
| Total gastrectomy* | 220 (0.0%) | 32 (0.0%) | 3 (0.0%) | 255 (0.0%) | 0.1517 |
| Resection of small intestine* | 7541 (1.2%) | 1747 (2.3%) | 330 (1.5%) | 9618 (1.3%) | <.0001 |
| Partial colectomy* | 19797 (3.2%) | 3923 (5.2%) | 861 (4.0%) | 24581 (3.4%) | <.0001 |
| Total colectomy | 924 (0.2%) | 359 (0.5%) | 106 (0.5%) | 1389 (0.2%) | <.0001 |
| Bowel obstruction* | 4198 (0.7%) | 790 (1.0%) | 172 (0.8%) | 5160 (0.7%) | <.0001 |
| Appendectomy* | 51907 (8.4%) | 716 (0.9%) | 79 (0.4%) | 52702 (7.4%) | <.0001 |
| Splenectomy* | 1239 (0.2%) | 262 (0.3%) | 31 (0.1%) | 1532 (0.2%) | <.0001 |
| Pancreatectomy* | 1056 (0.2%) | 190 (0.2%) | 23 (0.1%) | 1269 (0.2%) | <.0001 |
| Nephrectomy | 6344 (1.0%) | 480 (0.6%) | 63 (0.3%) | 6887 (1.0%) | <.0001 |
| Cystectomy | 12665 (2.1%) | 1496 (2.0%) | 384 (1.8%) | 14545 (2.0%) | 0.0031 |
| Cholecystectomy | 43485 (7.1%) | 2032 (2.7%) | 351 (1.6%) | 45868 (6.4%) | <.0001 |
| Hysterectomy | 61892 (10.1%) | 931 (1.2%) | 81 (0.4%) | 62904 (8.8%) | <.0001 |
| Lysis of abdominal adhesions | 18950 (3.1%) | 1732 (2.3%) | 303 (1.4%) | 20985 (2.9%) | <.0001 |
| Lobectomy* | 7043 (1.1%) | 489 (0.6%) | 42 (0.2%) | 7574 (1.1%) | <.0001 |
| Pneumonectomy* | 1607 (0.3%) | 112 (0.1%) | 12 (0.1%) | 1731 (0.2%) | <.0001 |
| Nephrectomy | 9281 (1.5%) | 1010 (1.3%) | 248 (1.1%) | 10539 (1.5%) | <.0001 |
| Prostatectomy | 7688 (1.3%) | 112 (0.1%) |  | 7800 (1.1%) | <.0001 |
| Oophorectomy | 6536 (1.1%) | 95 (0.1%) | 7 (0.0%) | 6638 (0.9%) | <.0001 |
| Salpingo-oopherectomy | 30383 (4.9%) | 751 (1.0%) | 55 (0.3%) | 31189 (4.4%) | <.0001 |
| Hysterectomy | 55154 (9.0%) | 772 (1.0%) | 44 (0.2%) | 55970 (7.9%) | <.0001 |
| Spinal vertebral repair | 6168 (1.0%) | 481 (0.6%) | 74 (0.3%) | 6723 (0.9%) | <.0001 |
| Discectomy | 777 (0.1%) | 29 (0.0%) | 4 (0.0%) | 810 (0.1%) | <.0001 |
| Spinal fusion | 3966 (0.6%) | 396 (0.5%) | 54 (0.2%) | 4416 (0.6%) | <.0001 |
| Below-knee amputation | 1055 (0.2%) | 1075 (1.4%) | 382 (1.8%) | 2512 (0.4%) | <.0001 |
| Above-knee amputation | 325 (0.1%) | 467 (0.6%) | 262 (1.2%) | 1054 (0.1%) | <.0001 |
| Metatarsal amputation | 1194 (0.2%) | 713 (0.9%) | 197 (0.9%) | 2104 (0.3%) | <.0001 |
| ORIF femur | 9396 (1.5%) | 5917 (7.8%) | 2478 (11.4%) | 17791 (2.5%) | <.0001 |
| Knee arthroplasty | 55215 (9.0%) | 2040 (2.7%) | 259 (1.2%) | 57514 (8.1%) | <.0001 |
| Hip arthroplasty | 46430 (7.6%) | 9313 (12.2%) | 3705 (17.0%) | 59448 (8.3%) | <.0001 |
| Ankle ORIF | 23734 (3.9%) | 1090 (1.4%) | 194 (0.9%) | 25018 (3.5%) | <.0001 |
| Rotator cuff repair | 7575 (1.2%) | 305 (0.4%) | 51 (0.2%) | 7931 (1.1%) | <.0001 |
| Fixation of FRU | 13387 (2.2%) | 846 (1.1%) | 141 (0.6%) | 14374 (2.0%) | <.0001 |
| Fixation of FTF | 10584 (1.7%) | 749 (1.0%) | 99 (0.5%) | 11432 (1.6%) | <.0001 |
| Cruciate ligament repair | 4153 (0.7%) | 35 (0.0%) | 1 (0.0%) | 4189 (0.6%) | <.0001 |
| GI endoscopic | 67752 (11.0%) | 25590 (33.6%) | 7497 (34.5%) | 100839 (14.1%) | <.0001 |
| Cystoscopy, TURP, TURBT | 47790 (7.8%) | 6683 (8.8%) | 2171 (10.0%) | 56644 (7.9%) | <.0001 |
| Cataract | 1784 (0.3%) | 153 (0.2%) | 81 (0.4%) | 2018 (0.3%) | <.0001 |
| Mastectomy and superficial | 81330 (13.2%) | 9307 (12.2%) | 2948 (13.6%) | 93585 (13.1%) | <.0001 |
| Bronchoscopy | 16344 (2.7%) | 6901 (9.1%) | 1631 (7.5%) | 24876 (3.5%) | <.0001 |
| Abdominal wall hernia repair | 31054 (5.1%) | 2966 (3.9%) | 557 (2.6%) | 34577 (4.9%) | <.0001 |

Abbreviations: HFRS – Hospital frailty risk score; CCI – Charlson comorbidity index; FRU – fracture, radius and ulna; FTF - fracture, tibia and fibular.
