# Supplementary material for: Beyond the revised cardiac risk index: Validation of the hospital frailty risk score in non-cardiac surgery
Source: PLoS One. 2022 Jan 19;17(1):e0262322. doi: 10.1371/journal.pone.0262322 (PMC8769314; doi:10.1371/journal.pone.0262322)
Supplement: S5 Table — (DOCX) [file pone.0262322.s005.docx]

**S5 Table. Rate of Major Cardiac Events (Death, Myocardial Infarction or Cardiac Arrest within 30-days of discharge) Stratified by RCRI, HFRS and Sex.**

|  |  | **Rates of MACE** | | | **p-value** |
| --- | --- | --- | --- | --- | --- |
|  |  | **Overall**  (n=712808) | **Female**  **(**n=392501) | **Male**  (n=320307) |  |
| **RCRI=0**  (n=389863) | Low HFRS  (n=348747) | 1139/348747  0.33%) | 492/182513,  0.27% | 647/166234  0.39% | <.0001 |
|  | Intermediate HFRS  (n=34092) | 434/34092  1.27% | 231/18615  1.24% | 203/15477  1.31% |  |
|  | High HFRS  (n=7024) | 78/7024  1.11% | 45/4275  1.05% | 33/2749  1.20% |  |
|  | Total HFRS  (n=389863) | 1651/389863  0.42% | 768/205403  0.37% | 883/184460  0.48% |  |
| **RCRI=1**  (n=271834) | Low HFRS  (n=240382) | 835/240382  0.35% | 319/150635  0.21% | 516/89747  0.57% | <.0001 |
|  | Intermediate HFRS  (n=24480) | 470/24480  1.92% | 211/12126  1.74% | 259/12354  2.10% |  |
|  | High HFRS  (n=6972) | 148/6972  2.12% | 72/3835  1.88% | 76/3137  2.42% |  |
|  | Total HFRS  (n=271834) | 1453/271834  0.53% | 602/166596  0.36% | 851/105238  0.81% |  |
| **RCRI=2**  (n=10819) | Low HFRS  (n=4129) | 114/4129  2.76% | 40/1302  3.07% | 74/2827  2.62% | <.0001 |
|  | Intermediate HFRS  (n=4472) | 212/4472  4.74% | 69/1656  4.17% | 143/2816  5.08% |  |
|  | High HFRS  (n=2218) | 114/2218  5.14% | 48/1041  4.61% | 66/1177  5.61% |  |
|  | Total HFRS  (n=10819) | 440/10819  4.07% | 157/3999  3.93% | 283/6820  4.15% |  |
| **RCRI ≥3**  (n=40292) | Low HFRS  (n=21663) | 458/21663  2.11% | 139/8251  1.68% | 319/13412  2.38% | <.0001 |
|  | Intermediate HFRS  (n=13092) | 417/13092  3.19% | 148/5470  2.71% | 269/7622  3.53% |  |
|  | High HFRS  (n=5537) | 217/5537  3.92% | 101/2782  3.63% | 116/2755  4.21% |  |
|  | Total HFRS  (n=40292) | 1092/40292  2.71% | 388/16503  2.35% | 704/23789  2.96% |  |
