# Supplementary material for: Beyond the revised cardiac risk index: Validation of the hospital frailty risk score in non-cardiac surgery
Source: PLoS One. 2022 Jan 19;17(1):e0262322. doi: 10.1371/journal.pone.0262322 (PMC8769314; doi:10.1371/journal.pone.0262322)
Supplement: S6 Table — (DOCX) [file pone.0262322.s006.docx]

**S6 Table. NRI Analysis.**

|  | **NRI for events** | **NRI for non-events** | **NRI** | **p-value** |
| --- | --- | --- | --- | --- |
| **Prolonged Hospital Stay** | -0.422 | 0.627 | 0.205 | <0.001 |
| **In-hospital Mortality** | 0.320 | 0.634 | 0.954 | <0.001 |
| **30-Day ER/Readmission** | -0.504 | 0.635 | 0.130 | <0.001 |
| **30-Day Mortality** | -0.125 | 0.450 | 0.325 | <0.001 |
| **30-Day MACE** | -0.175 | 0.288 | 0.133 | <0.001 |
| **1-Year ER/readmission** | -0.481 | 0.682 | 0.201 | <0.001 |
| **1-Year Mortality** | -0.077 | 0.434 | 0.357 | <0.001 |
| **1-Year MACE** | -0.105 | 0.450 | 0.354 | <0.001 |
