# Supplementary material for: Beyond the revised cardiac risk index: Validation of the hospital frailty risk score in non-cardiac surgery
Source: PLoS One. 2022 Jan 19;17(1):e0262322. doi: 10.1371/journal.pone.0262322 (PMC8769314; doi:10.1371/journal.pone.0262322)
Supplement: S7 Table — (DOCX) [file pone.0262322.s007.docx]

**S7 Table. AUROC Analysis.**

|  | **AUROC of Gold** | **AUROC of**  **Gold +HFRS** | **AUROC Improvement** | **p-value** |
| --- | --- | --- | --- | --- |
| **Prolonged Hospital Stay** | 0.6583 | 0.6719 | 0.0136 | <0.001 |
| **In-hospital Mortality** | 0.8709 | 0.9006 | 0.0296 | <0.001 |
| **30-Day ER/Readmission** | 0.5858 | 0.5951 | 0.0093 | <0.001 |
| **30-Day Mortality** | 0.8780 | 0.8881 | 0.0100 | <0.001 |
| **30-Day MACE** | 0.8667 | 0.8730 | 0.0063 | <0.001 |
| **1-Year ER/readmission** | 0.6079 | 0.6229 | 0.0151 | <0.001 |
| **1-Year Mortality** | 0.8675 | 0.8787 | 0.0112 | <0.001 |
| **1-Year MACE** | 0.8535 | 0.8620 | 0.0085 | <0.001 |
